# Using facial reaction analysis and machine learning to objectively assess the taste of medicines in children

**DOI:** 10.1371/journal.pdig.0000340

**Published:** 2024-11-20

**Authors:** Rabia Aziza, Elisa Alessandrini, Clare Matthews, Sejal R Ranmal, Ziyu Zhou, Elin Haf Davies, Catherine Tuleu

**Affiliations:** 1 Aparito Ltd, Wrexham, United Kingdom; 2 University College London, School of Pharmacy, London, United Kingdom; Indiana University Purdue University at Indianapolis, UNITED STATES OF AMERICA

## Abstract

For orally administered drugs, palatability is key in ensuring patient acceptability and treatment compliance. Therefore, understanding children’s taste sensitivity and preferences can support formulators in making paediatric medicines more acceptable. Presently, we explore if the application of computer-vision techniques to videos of children’s reaction to gustatory taste strips can provide an objective assessment of palatability. Children aged 4 to 11 years old tasted four different flavoured strips: no taste, bitter, sweet, and sour. Data was collected at home, under the supervision of a guardian, with responses recorded using the Aparito Atom app and smartphone camera. Participants scored each strip on a 5-point hedonic scale. Facial landmarks were identified in the videos, and quantitative measures, such as changes around the eyes, nose, and mouth, were extracted to train models to classify strip taste and score. We received 197 videos and 256 self-reported scores from 64 participants. The hedonic scale elicited expected results: children like sweetness, dislike bitterness and have varying opinions for sourness. The findings revealed the complexity and variability of facial reactions and highlighted specific measures, such as eyebrow and mouth corner elevations, as significant indicators of palatability. This study capturing children’s objective reactions to taste sensations holds promise in identifying palatable drug formulations and assessing patient acceptability of paediatric medicines. Moreover, collecting data in the home setting allows for natural behaviour, with minimal burden for participants.

## Author summary

When formulating medicines for children, understanding the taste profile is crucial to ensure they are not excessively unpleasant. In this study, we assessed if facial reactions in response to taste stimuli can be used to easily measure children’s feelings about the taste. We recorded videos of children trying four different taste strips and analysed their facial expressions in response to each one. The strips had different flavours: bitter, sweet, sour, and no taste. We also asked the children to rate each strip on a scale from 1 to 5. We collected data from 64 children. The results confirmed that children generally like sweet tastes and do not like bitter ones. Their opinions on sour taste varied. Moreover, we found that specific facial reactions, like changes in their eyebrows and mouth corners were good indicators of taste perceptions. The analysis of facial expressions can help formulators make medicines for children taste better. By objectively measuring how children feel about the taste of medicines, we can create more acceptable medicines for them. The collection of this data at home ensured children were in their comfortable environment, making it easier for them to take part in the study.

## Introduction

The concept of patient acceptability is gaining progressive relevance in the development of paediatric dosage forms. Acceptability is defined as the overall ability and willingness of the patient and caregiver to use a medicinal product as intended or authorised and it is determined by characteristics of the user (age, ability, disease type) and of a medicinal product (e.g. palatability, swallowability, appearance) [1].

Thus, acceptability can have a significant impact on the patient’s adherence and consequently on the safety and efficacy of a product. For this reason, the European Medicines Agency (EMA) has repeatedly emphasised the importance and incentive to assess the acceptability of formulations for paediatric use, including in its 2006 Reflection Paper [2] and 2014 guideline on pharmaceutical development of medicinal products for children [1]. Consequently, in recent years, there has been an increased emphasis on conducting studies examining factors affecting the acceptability of medicine in children.

For orally administered drugs, palatability is key in determining patient acceptability and treatment compliance [1]. Palatability is defined as the overall appreciation of a medicinal product in relation to its smell, taste, aftertaste, and texture (i.e. feeling in the mouth) [1]. Specifically, taste is frequently reported to be a common reason for non-compliance among children [3]. Thus, regulatory agencies strongly encourage the inclusion of acceptability testing, including palatability assessment, as an integral part of the product development and embedded within clinical programs in the target patient population [3].

Several methodologies for palatability assessment in children are available, and they have been largely reviewed [3–7]. These methodologies should be age-appropriate and, depending on the age of the child, may involve collecting data from patients and/or caregivers [4]. The selection of appropriate taste assessment methodologies is determined by the cognitive capacity of the child, and until now, there is no methodology validated for accuracy and reliability with any particular age group [3,6]. The facial hedonic scale is considered the gold standard for drug palatability testing in children [8]; however, this scale cannot be used in very young children or in those with communication issues, cognitive impairments, and/or developmental delays.

The EMA reflection paper defines four key criteria for palatability assessment in children: 1) the test should be short, 2) the test must be intrinsically motivating and “fun” to do, 3) the procedure should be as easy as possible, 4) the number of variants to be tested should be limited to a maximum of four. However, the reflection paper does not aim to provide any scientific, technical, or regulatory guidance [2]. This suggests the opportunity for the development of more objective quantitative technological advancements such as the use of high throughput systems [https://www.opertechbio.com/technology], facial electromyography [9], electrogustometry [10], or facial recognition technology [8,11] in palatability assessment [3].

Observation of children’s facial reactions after exposure to a taste stimulus is not new. Some of the earliest investigations on taste in children consisted of videotaping infants and then characterising their oromotor reflexes when taste stimuli were placed on the tongue or in the oral cavity [12–15]. In 1988, Oster and Rosenstein [15] developed a method for describing orofacial responses by using the Ekman and Friesen’s [16] anatomically based Facial Action Coding System (FACS). FACS virtually decomposes any facial expression into its constituent action units (AUs). Video records are analysed in slow motion to quantify the actual number of affective reactions infants express to a taste stimulus, as a measure of valence and intensity [7,17]. The advantage of using this method is that it can be used in non-verbal children such as infants. However, the analysis of video images requires trained individuals to establish reliability across scores and it can be subjective [18]. Moreover, this method is time-consuming and costly [7].

The recent and rapid advent of artificial intelligence (AI) and machine learning (ML) techniques in our daily lives has enabled computers to perform automatic facial expression recognition (FER) accurately and efficiently. This has resulted in the establishment of human-computer interaction systems with good intelligence and interaction performance [19]. Currently, FER can be executed via two methods: traditional machine learning or deep learning [19]. The traditional machine learning method involves image pre-processing, feature extraction, and classification, whereas the deep neural network method omits the manual feature extraction step and utilises the convolution operation for feature extraction [19,20]. However, deep learning methods typically require a significantly larger training data set than traditional ML techniques.

FER has attracted the attention of numerous researchers, showing potential for application in a wide range of fields [20], including healthcare, education, personal identification verification, public safety, human-computer interaction, and retailing [19,20].

In the medical field, FER has been utilised for various applications, such as pain detection [21,22], studying mental disorders like autism [23,24], attention-deficit/hyperactivity disorder (ADHD) [25], schizophrenia, or depression [26]. Other studies have explored foetal facial expressions in relation to brain development [27], or changes in FER associated with diseases like Parkinson’s [28,29].

In recent years, FER has been increasingly used to assess consumer preferences and acceptance of food and drinks [30], yielding promising results [31–37]. The human face can convey appreciation or dislike while eating and drinking [32] serving as a cue to determine whether someone likes a particular taste or not, as it provides rich and spontaneous data in terms of facial expressions [32].

Despite similarity between this field of investigation and the assessment of the taste of medicines, only a few previous studies have attempted to apply FER in the context of medication palatability. Moreover, a study investigating children’s emotion recognition using various deep-learning models with explainable AI has underscored the differences in facial construction between children and adults [38]. It has been noted that children exhibit emotions in unique ways that do not always align with the standardised facial expressions observed in adults [38]. Consequently, there is a growing recognition of the necessity to conduct specialised studies focusing on children.

Kearns et al. [39] undertook a prospective, pilot study to assess the feasibility of using facial recognition technology to assess drug palatability in 10 children aged between 7 to 17 years. Although the qualitative assessment of the facial recognition data demonstrated the ability to discriminate between bitter and sweet tastants, their facial recognition software (Noldus FaceReader 7) and approach showed some limitations in discriminating taste profiles and highlighted that further refinement was required before this methodology can be applied more widely [8]. The Noldus FaceReader 7 software measures the intensities of a predefined set of emotions e.g., happy, angry, disgusted etc., on a frame-by-frame basis.

Similarly, Peng et al.[40], used the same software to compare the palatability of two preparations of carbocysteine among healthy children aged 2 to 12 years. The palatability assessed by emotional valences was performed using a facial action coding system by FaceReader, which reflected the quantification of emotions; a positive value represents a positive emotion, and a negative value represents a negative emotion [40].

Presently, we refine the work of Kearns’ group [39], to explore if the application of computer-vision techniques to videos of primary school children’s reaction to gustatory taste strips can provide an objective assessment of taste. Our methodology uses pose estimation for facial recognition analysis, which allows access to the raw movements of facial features, rather than through the lens of emotional reactions.

In tasting, facial expressions may not always directly reflect inner emotional states but rather serve as a spontaneous motor response to flavours or tastes. Therefore, facial analysis for emotion classification (e.g., Action Units) may not be directly applicable to taste liking. For example, a person may display facial Action Units (AU) corresponding to disgust expression (e.g., lip corner depressor, or nose wrinkle) when tasting lemon juice, yet, this does not necessarily indicate that he/she dislikes the taste. Additionally, beside the appearance of taste-induced facial expressions, subtle dynamic information hidden in such expressions is important [32].

Finally, our study was conducted in a domiciliary setting to allow for natural behaviour, with minimal burden for participants.

## Materials and methods

### Participants

Participants were children aged between 4 to 11 years old recruited from a primary school in London, United Kingdom, and their adult caregiver. Prior to the study, all participants received an information sheet with the study details. Participants and their caregivers signed informed consent and assent forms respectively if they were willing to participate in the study. The study was approved by the UCL research ethics committee (REC) 4612/029.

As this was an exploratory study, no formal requirements were put on sample size. All pupils at a school of 840 were invited to join the study.

### Study design and procedures

This study was a single blind taste assessment. After consent, all participants were provided with a study pack for the taste evaluation to be completed at home. The pack contained the study instructions, four taste strips and instructions on how to download the Aparito app (Atom5) on their smartphone. Atom5 is a secure, encrypted and password protected platform (ISO 13845, ISO 27001, Cyber Essential Plus, CREST tested and ePrivacyApp awarded by ePrivacyseal GmbH) designed to collect digital endpoint data. It has been used for remote monitoring across a wide range of adult and paediatric disease areas. Commercially available Burghart (or ODOFIN) taste strips (MediSense, Groningen, The Netherlands) were used in this study. The taste strips are made of filter paper impregnated with different solutions containing substances found in food and drinks. They are used in clinical and research contexts as a validated procedure to investigate taste ability/gustatory sensitivity in both children and adults [41]. One of the strips was blank with no tastant, one strip was bitter (0.006 g/mL of quinine hydrochloride), one was sweet (0.4 g/mL of sucrose), and one sour (0.3 g/mL of citric acid). Each taste strip was individually repackaged into coloured Mylar foil bags for blinding purposes: blank in a white coloured foil bag, bitter in yellow, sweet in green and sour in purple.

**Fig 1:**
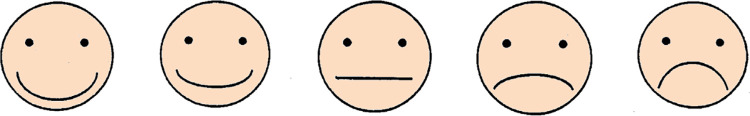
A 5-point hedonic smiley face scale that was used in the study.

After receiving the study pack, participants were asked to input their age and sex on the app. Then, instructions guided the participants through each step of the study. Children were instructed to place one strip on the middle of their tongue, close their mouth and test the sample for 10 seconds before removing the strip. The adult caregiver used their smartphone video to record the facial reaction of the child as they tasted the strip. After removing each strip, the children were asked to rate the sample on a 5-point hedonic smiley face scale, where 1 corresponded to a sad face, indicative of dislike and 5 to a happy face indicating the liking of the taste of the strip, Fig 1. Finally, children were invited to provide their feedback about the tasting experience through an open response question.

Instructions indicated to test the blank control strip first so that participants could practise the correct use of the strip, the video recording, and how to record their responses in the app. The other three taste strips were tested in three different sequences as indicated by the foil bag colour (sweet, sour, bitter—sour, bitter, sweet—bitter, sweet, sour) and children were randomly assigned to one of the three sequences by the app. To avoid any anticipation and bias in their responses, participants were blinded to the taste of the strip they were tasting and were instructed to taste each strip sequentially from each coloured foil bag. Children were invited to take some water to help remove any residual taste between each sample.

All data recorded within the app was transferred onto the Atom5 software platform and stored securely on Microsoft Azure. All data were collected and stored in accordance with the Data Protection Legislation 2018 and General Data Protection Regulation (GDPR) 2018.

### Statistical analysis

For each taste strip, the rank rating of the hedonic scale was analysed to see if any difference could be appraised between different age groups, sex, and order of strip testing. Also, differences in rank rating between different tastes were assessed. The Kruskal-Wallis H test was used for the analysis and the IBM SPSS Statistics software platform (IBM Corp. Released 2021. IBM SPSS Statistics for Windows, Version 28.0. Armonk, NY: IBM Corp) was used with the limit of statistical significance set at α = 0.05.

### Machine learning for pose estimation

#### Pose estimation framework

We used a CNN (Convolutional Neural Network) based, open-source machine learning (ML) framework, MediaPipe [42]. MediaPipe is a perception pipeline builder that offers different pose estimation components, including face detection, face landmarks, hands, body, and object tracking. In this study, we used the Face-Landmark-v1 component. This component is based on the BlazeFace model [43] for face detection, and estimates 368 3D face landmarks per frame. Kartynnik et al.[44] reported an accuracy of 3.96% of the full Face-Landmark model, calculated as the mean absolute distance between the predictions and the ground truth vertex locations, normalised by interocular distance.

#### Data pre-processing

The video data were filtered on two levels: frame level and facial landmark level.

First, for each individual video, we identified which frames to include in the analysis. We defined the tasting task as the sequence of frames per video from when the taste strip was inserted in the subject’s mouth, until just before the action of removing said strip. The cleaning process included reviewing the videos and manually identifying the beginning and end times of the tasting task. Importantly, we noticed that some subjects reacted after the taste strip was removed from the mouth. Therefore, we also identified a post-tasting section that started from the moment after the strip was removed from the mouth, until the end of the video or until the subject was distracted by something else (e.g., if the child starts talking to someone else in the room or turns away and moves out of the frame).

**Fig 2:**
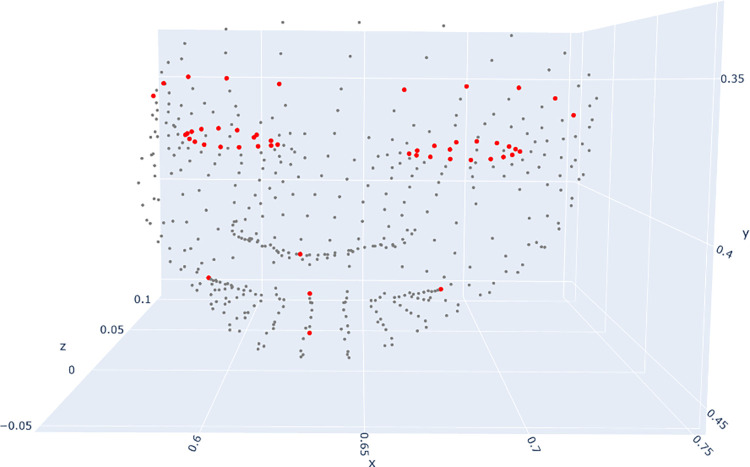
Landmarks identified to be included in the analysis (red) plotted against all landmarks estimated by MediaPipe (grey).

Secondly, we identified 53 key facial landmarks out of the 368 landmarks extracted by MediaPipe. These landmarks were carefully selected to outline the main visible facial features, as illustrated in Fig 2. The geometric calculations involving these landmarks are presented in the subsequent section. By focusing our analysis on these critical regions, we aimed to capture the most relevant facial characteristics while minimising extraneous data and noise. Data used in this study are available at: https://git.aparito.tech/rabiaa/tasty_public_dataset.

#### Facial feature aggregation

The extracted landmarks were aggregated into the following measures:

- Eyebrow elevation: left and right variants calculated as the Euclidean distance between the median of the eyebrow landmarks and the nose tip landmark.
- Eyebrow tilt: left and right variants computed as the angle between the fitted line through the five eyebrow landmarks and the line connecting the inner eye corners.
- Eyebrow shape: left and right variants determined by the angle between the lines connecting the middle eyebrow landmark with the left and right eyebrow endpoints.
- Palpebral aperture: left and right variants representing the surface area delineating the eye region.
- Lip elevation: upper and lower variants measured as the Euclidean distance between the middle lip landmark and the nose tip.
- Mouth corner: left and right variants calculated as the Euclidean distance between the mouth corner landmark and the nose tip.

These measures are a subset of those used in a study applying similar ML techniques to assess facial bradykinesia in patients with Parkinson’s disease [29]. The measures provide a practical means to transfer the facial areas of interest into quantifiable inputs to a ML model. A number of measures used in Novotny et al., 2022 [29] were specific to the patient population, disease and symptoms studied (e.g. cheek surface variability and jaw elevation/depression) and considered not relevant to the current study. Therefore, these were omitted. The subset included was selected as related to the facial features most often used in studies linking facial expressions with taste, i.e. eyes, eyebrows and mouth [45–47]. While MediaPipe can estimate pupil location, it does not provide data on pupil size, so we were unable to include pupillary response. Aside from lip elevation, all measures were calculated for the left and right side of the face independently as asymmetry in facial expressions has been reported broadly across studies into emotional response [48–50]. The description of left and right is from the point of view of the observer of the video. To account for variations in the landmark coordinates caused by factors other than facial reactions (e.g., the distance and angle of the camera changes over time as the person moves their head), all distance measures were scaled using a reference: the Euclidean distance between the right inner eye corner and the nose tip.

The above measured features were applied on each frame, irrespective of whether the facial expression represented a baseline (neutral), a semi-altered (transitioning) or most altered (peak state). This comprehensive approach was adopted because identifying the frame with the most altered expression can be challenging, as facial expressions evolve over time and may not manifest simultaneously across all facial regions. By including all frames, we aimed to capture the dynamic nature of facial expressions and ensure that no relevant information was overlooked.

## Results

### Data description and participants’ demographics

A total of 250 participants agreed to take part in the study and received a study pack at home. Of these, 40 participants completed all the hedonic ratings and video recordings, and 24 participants completed all the hedonic scales but did not record one or more videos. Thus, the data analysis was performed with data from 64 participants; Table 1 reports the number of hedonic scales and videos recorded per taste strip.

**Table 1:** Total number of hedonic scale ratings and videos analysed per each taste.

Taste of the strip	No. of hedonic scales	No. of videos
Neutral	64	51
Sour	64	46
Sweet	64	54
Bitter	64	46
Total	256	197

Children in the study were aged between 5 to 11 years, with a median age of 8.5 years (SD 1.46), Table 2, and there were slightly more females (n = 36) than males (n = 28).

**Table 2:** Age distribution of the children participating in the study.

Age (years)	Frequency (%)
5	1 (2%)
6	3 (5%)
7	10 (16%)
8	18 (28%)
9	13 (20%)
10	11 (17%)
11	8 (13%)

### Hedonic scale results

The ratings of the hedonic scale for each taste strip were analysed by sex, age, and randomisation order of the strips to assess if any difference between groups existed. The analysis by sex showed that there were no significant differences between boys and girls in terms of hedonic responses to the taste of each strip (χ2(2) = 0.46, p = 0.50 for the blank (control) strip, χ2(2) = 1.50, p = 0.23 for the sour strip, χ2(2) = 2.32, p = 0.13 for the sweet strip, and χ2(2) = 2.30, p = 0.13 for the bitter strip). Different ages also showed similar ranking scores for each taste strip (χ2(2) = 2.04, p = 0.92 for the control strip, χ2(2) = 4.19, p = 0.65 for the sour strip, χ2(2) = 3.92, p = 0.69 for the sweet strip, χ2(2) = 1.99, p = 0.92 for the bitter strip). Similarly, changing the order of how the taste strips were assessed did not alter the rating of each taste strip (χ2(2) = 0.64, p = 0.73 for the sour strip, χ2(2) = 0.41, p = 0.82 for the sweet strip, χ2(2) = 0.80, p = 0.67 for the bitter strip).

**Fig 3:**
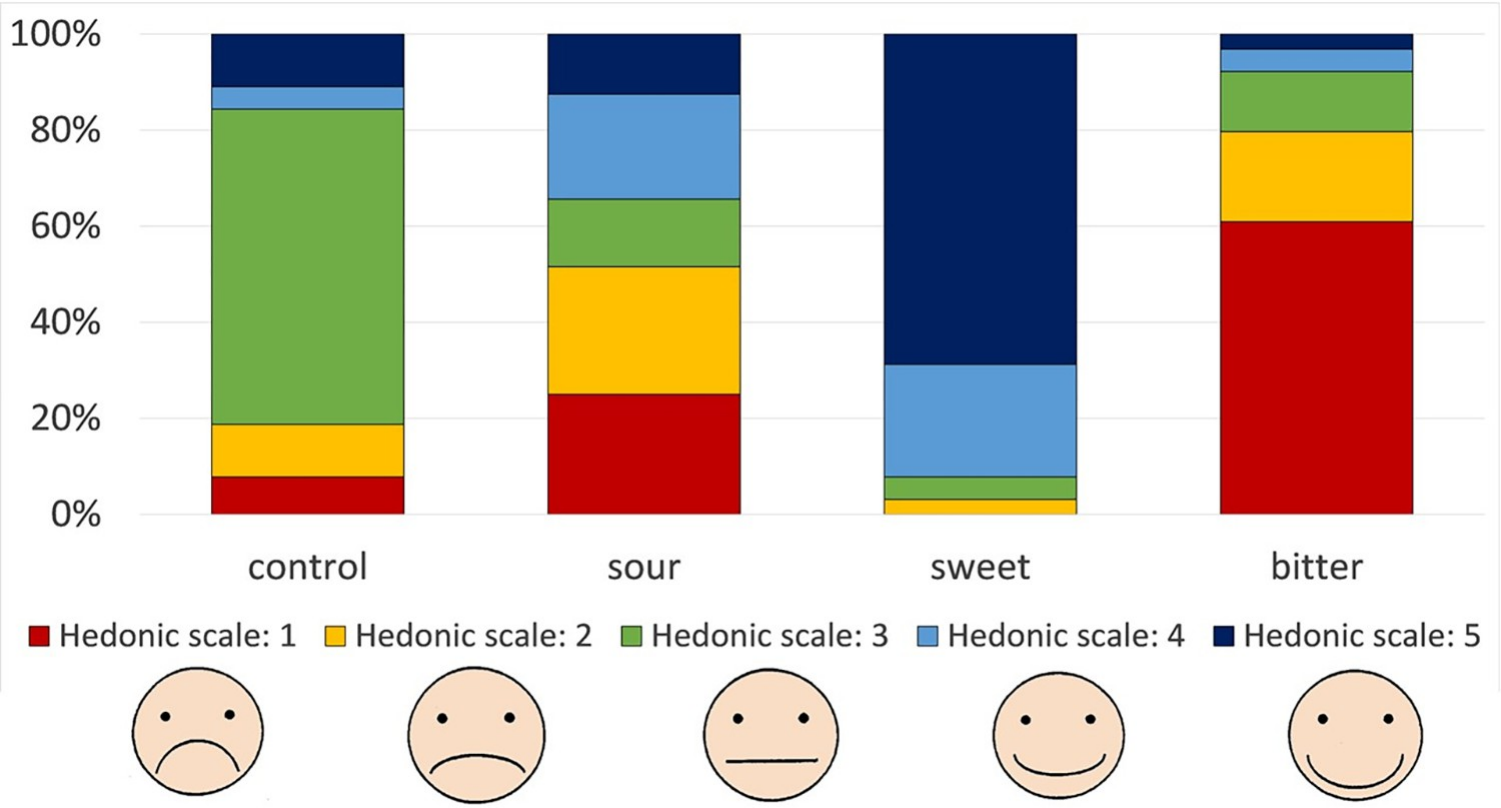
Participants’ hedonic scale rating for each taste strip, where 1 corresponds to not liking the taste (sad face), and 5 corresponds to liking the taste of the strip (happy face).

Instead, a statistically significant difference in ratings emerged across the different tastes (χ2(2) = 124.62, and p = 0.001), with the following predominant scores observed for each taste strip: 3 for the blank control strip, 1 for the bitter strip, 5 for the sweet strip, and various scores for the sour strip. These results indicate that the hedonic scale elicited expected results: children like sweetness, dislike bitterness and have varying opinions for sourness, Fig 3.

### Facial measures

**Fig 4:**
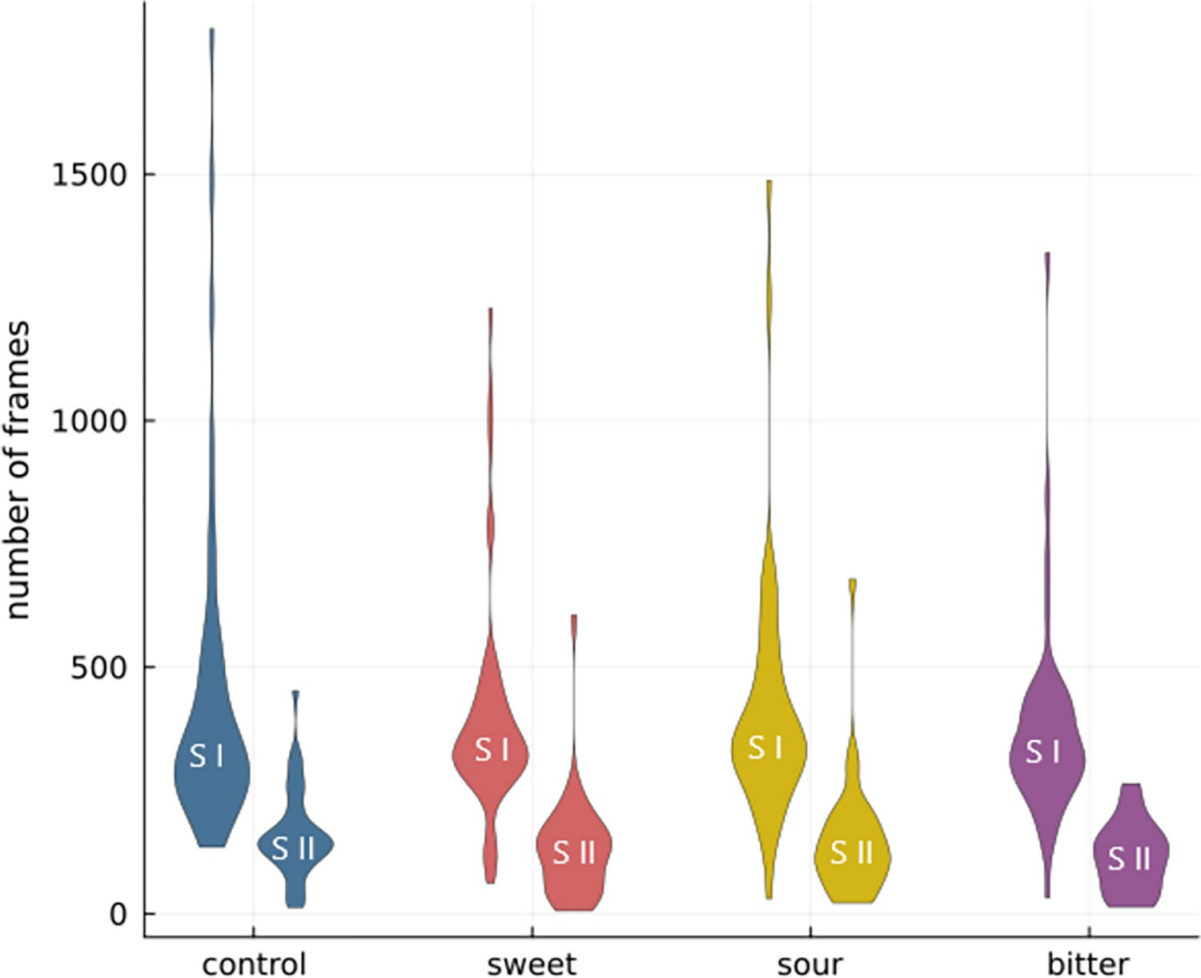
Number of frames processed with MediaPipe per taste and section: during the tasting (SI) and post tasting (SII).

Facial landmark coordinates were extracted for each frame captured during or after the tasting task. A total of 97.4% of frames (accounting for 95,561 total frames) were successfully processed using the MediaPipe framework. Failures to process some frames were due to subjects turning away from the camera or covering their face. Fig 4 shows the distribution of processed frames per video, during and after the tasting, grouped by taste.

**Fig 5:**
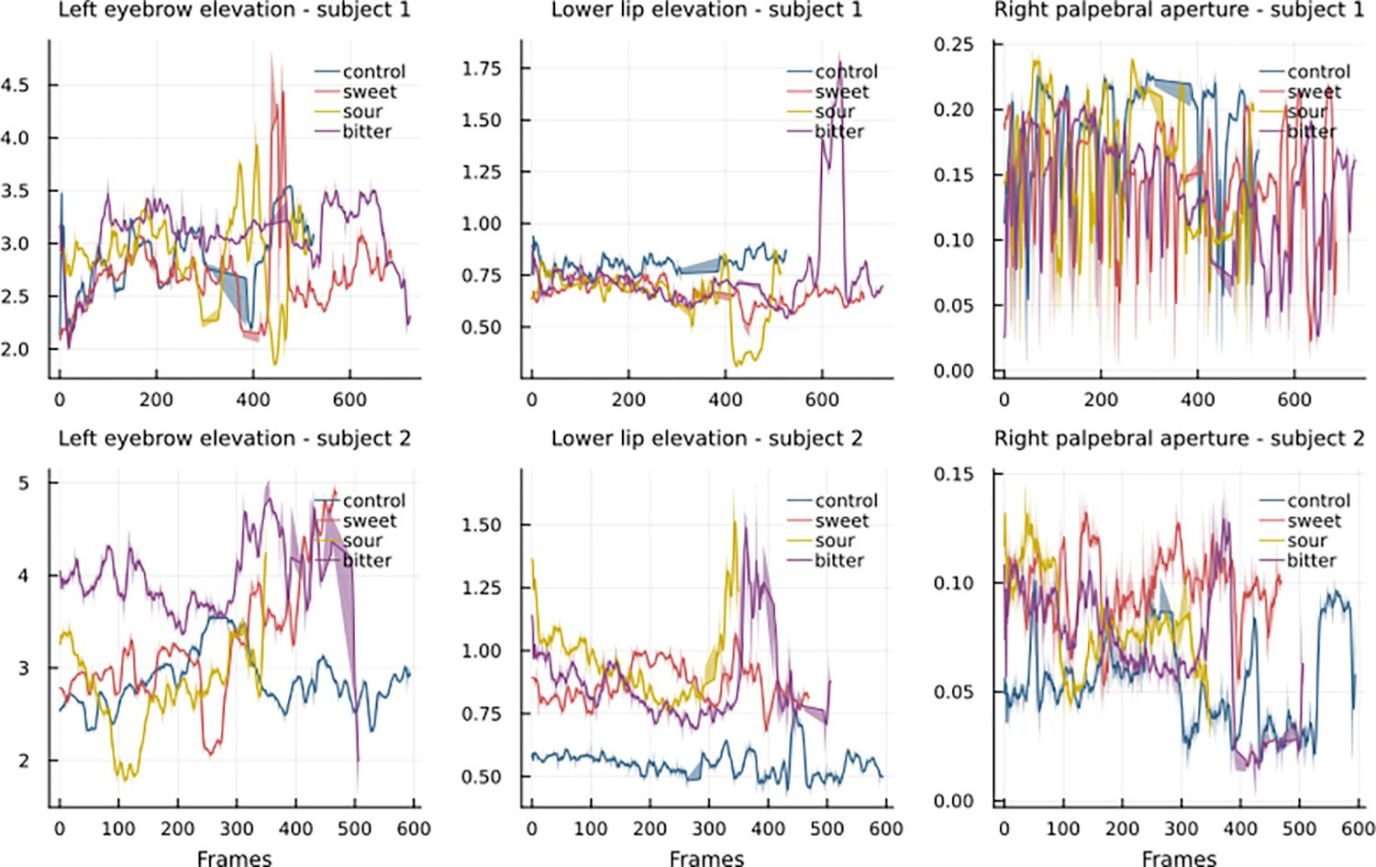
Sample of rescaled measures of two subjects.

Using the extracted coordinates, we aggregated the landmarks into facial feature measures. A sample data of two subjects (four videos per subject: one for each taste) is depicted in Fig 5 in a 5-frame moving average of three measures: the elevation of the left brow, the elevation of the lower lip, and the palpebral aperture of the right eye.

For each subject the following measures are presented: left eyebrow elevation, lower lip elevation, right palpebral aperture. The Figure shows the variation of the measures over time (represented by the x-axis and measured in frame number) per tasting (including both during and post tasting sections).

An ANOVA analysis was conducted to examine the effects of the taste categories (control, sweet, sour, bitter) and hedonic scores (ratings of the liking), on each of the measured variables (eyebrow elevation, mouth corner, etc.). The ANOVA results revealed statistically significant main effects of the taste and hedonic scores on the dependent measures (p < 0.05), indicating that the variability in these measures was significantly different across the different taste categories and levels of hedonic ratings.

**Fig 6:**
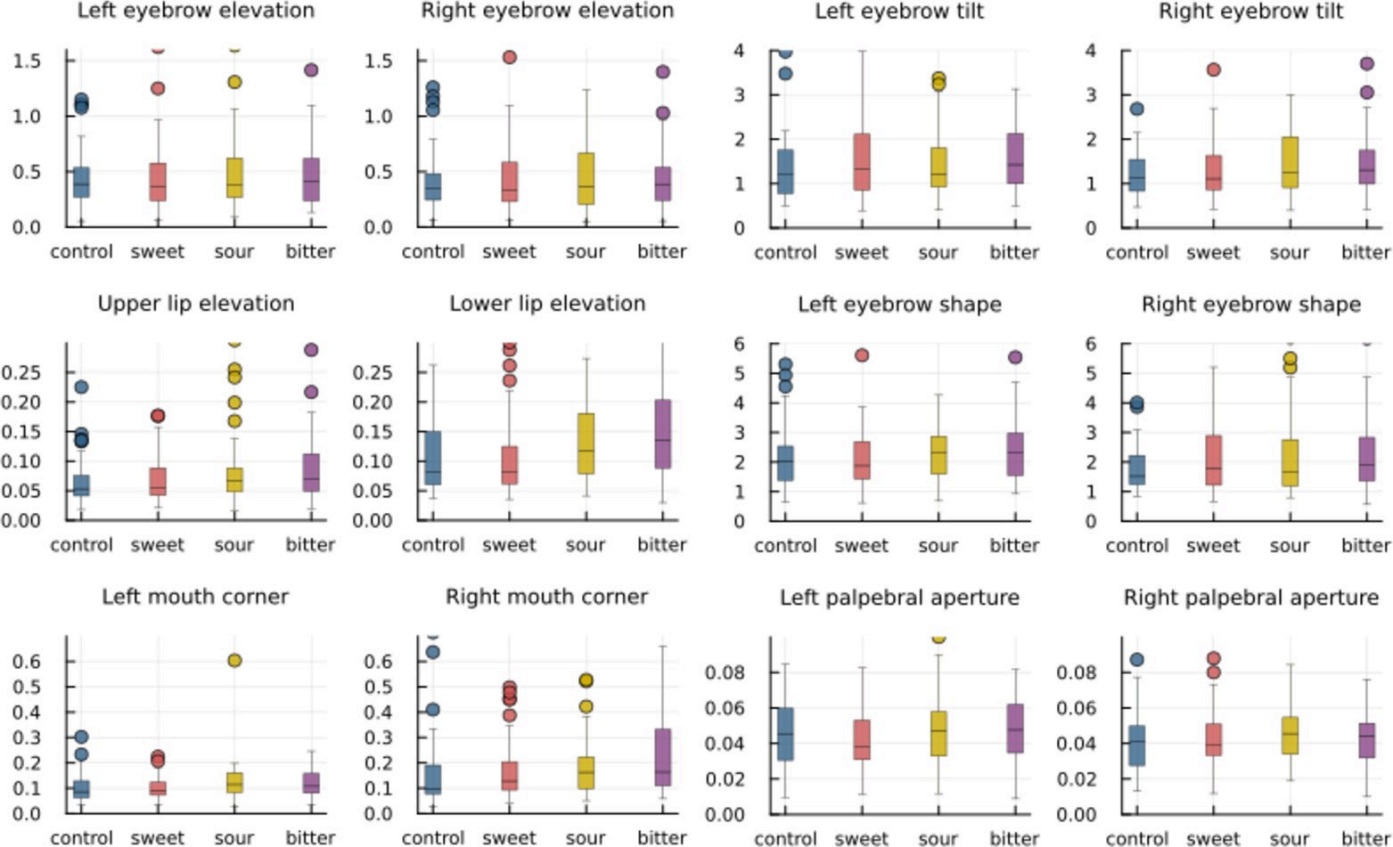
Box plots of the standard deviation of the rescaled measures, grouped by taste.

**Fig 7:**
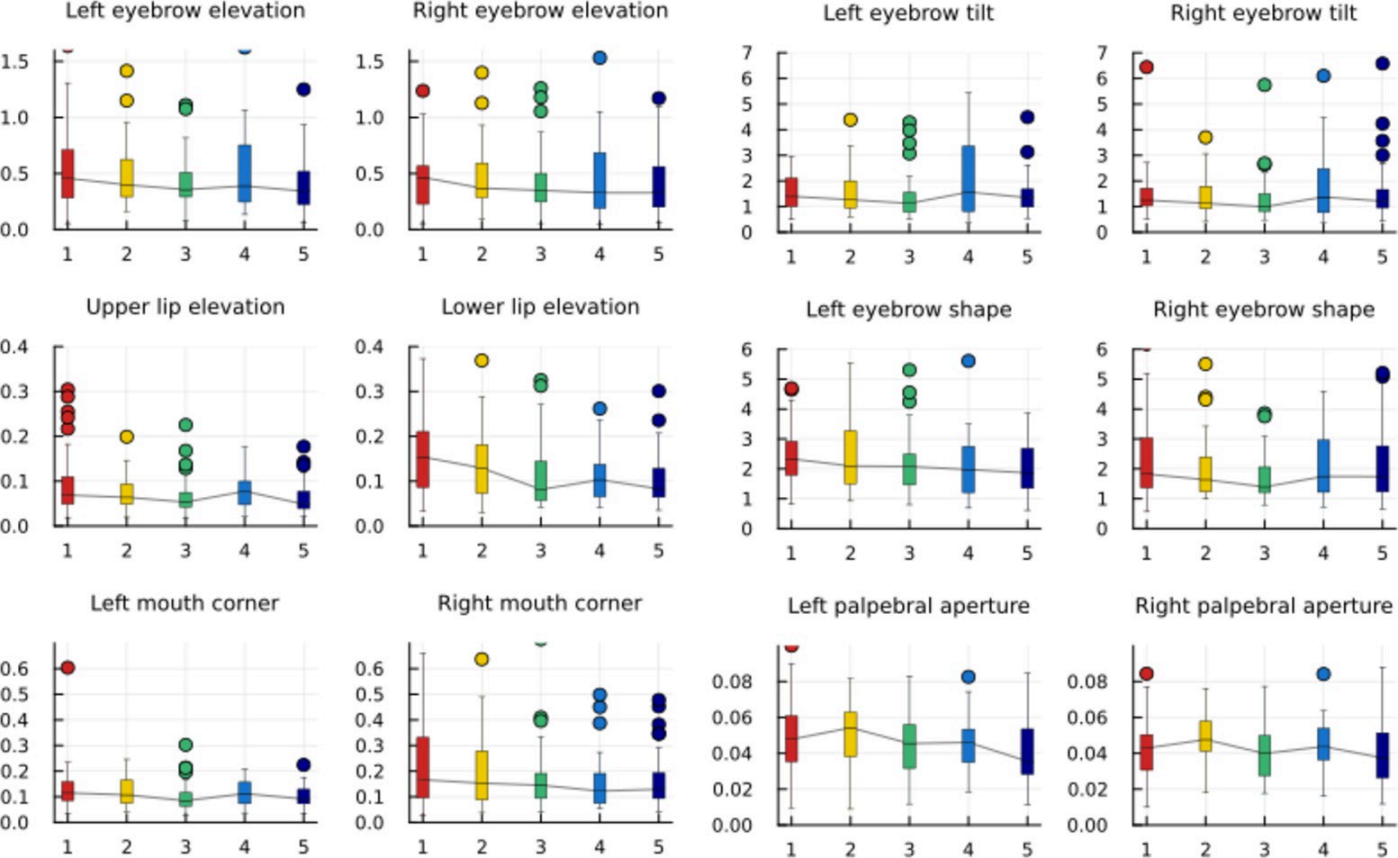
Box plots of the standard deviation of the rescaled measures, grouped by hedonic scores.

We then calculated the standard deviation of the rescaled measures for all subjects, including all their videos that passed quality checks. We plotted this, grouped per taste and hedonic score, in Figs 6 and 7.

**Fig 8:**
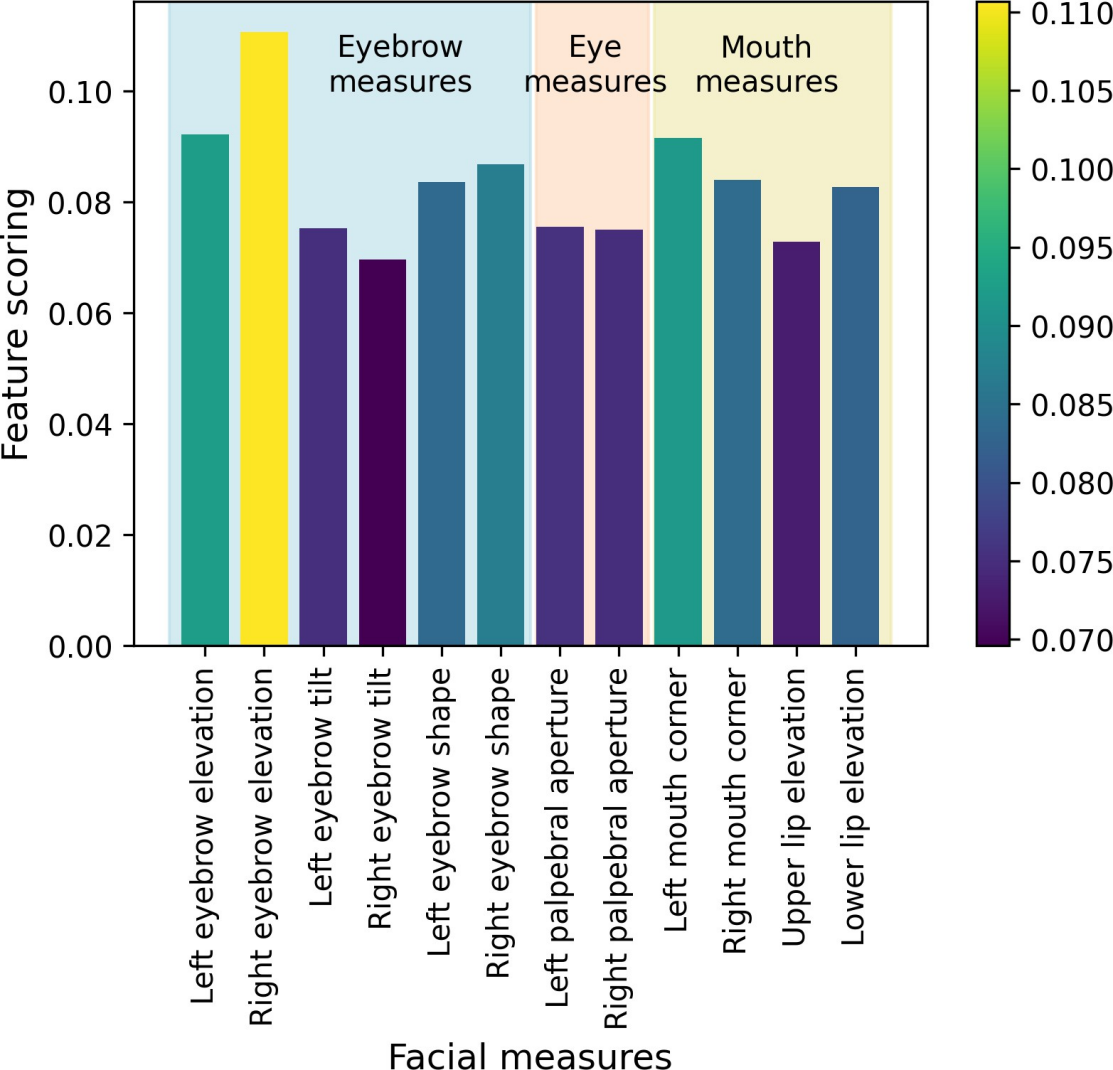
Ranking of the importance of the facial measures using an Extra-Trees classifier.

Moreover, we ran an analysis that determines which facial measure(s) were most predictive of palatability. We used an Extra-Trees classifier, which was trained with 200 trees in the forest, using the square root of the total number of features for splitting, a minimum of 5 samples for splitting internal nodes, and a minimum of 2 samples per leaf node, to rank the importance of each measure. The "Right eyebrow elevation" measure was found to be the most important, followed by the “Left eyebrow elevation” and the “Left/Right mouth corner”, Fig 8. To test the stability of the rankings, we also performed Principal Component Analysis (PCA) on the facial measures. While the rankings from PCA were slightly different, with "Left eyebrow elevation" being the most important followed by "Right eyebrow elevation" and "Upper lip elevation", the top-ranked features were generally consistent with the Extra-Trees classifier results, indicating the robustness of the findings.

## Discussion

The present study sought to investigate facial reactions to different tastes in children, with the aim of identifying key indicators of palatability perceptions. While this methodology may be particularly useful for assessing taste reactions in populations unable to self-report their reactions, such as very young children or those with communication or cognitive impairments, this study was conducted in ‘verbal children’. This was necessary to pilot and test the approach in a population capable of self-reporting their taste perceptions. This step is essential for calibrating and validating the new method. Only after refining and successfully validating this method in self-reporting children we may be able to proceed to test it in other populations.

Two previous studies have assessed the use of facial recognition technology to evaluate palatability in the context of medicinal products in children [39,40]. However, the main differences of our study compared to the previous studies are as follows. Firstly, our study was conducted in a domiciliary setting rather than in a standardised laboratory environment. While this approach can lead to reduced video quality and compliance with the instructions, our study showed that it is feasible, with the advantage of posing a minimal burden for participants.

Secondly, the methodology applied in our study differed from that of Kearns’ and Peng’s study [39,40] as we applied pose estimation for facial reaction analysis, assessing the raw movements of facial features rather than classifying the facial expressions in emotional reactions. This allowed us to specifically and directly assess reactions to taste stimuli without the need to translate them into emotions which can be biased by aspects other than the taste. Moreover, Kearns’ study further grouped reactions into positive and negative, based on the labels assigned to the reactions. In this current study we were interested to see whether using the raw facial features could elicit improved results.

Finally, taste strips were used instead of liquids to measure the palatability. The advantage of using taste strips rather than solutions is that the latter pose inherent quality and safety related challenges to taste testing, given their bulkiness and thus difficulty of storage and transport, as well as their swallowing risk, particularly if used by children [51]. This taste strip methodology has also been successfully implemented in this age group in another recent study [52].

This study showed that taste strips were easy to use and results from the hedonic scale showed that expected results were elicited: children like sweetness, dislike bitterness and have varying opinions for sourness. Previous studies have reported that children’s liking of sweet and disliking of bitter reflect their basic biology [53]. The appreciation for sourness seems to be influenced by children’s food habits and there are various experiential factors that can influence flavour preferences during childhood [54,55].

In the 197 videos available, the relative proportions of frames per taste were as follows: 28.29% control, 26.15% sweet, 24.40% sour, and 21.16% bitter. The lower value for bitter was due to less adherence to the 10 seconds minimal duration of the video.

This balanced distribution of processed frames across the different tastes enabled a comprehensive evaluation of facial reactions.

Overall, our findings demonstrate a clear signal of a reaction to taste, but also highlight the complexity and variability of facial reactions in response to different tastes.

For instance, Fig 5 illustrates how the bitter taste resulted in a higher lower lip elevation, while the sweet taste elicited a higher left eyebrow in both subjects. However, these signals were not consistent across all measures and subjects, indicating the complex nature of taste perception. Moreover, our results suggest the existence of an inter-individual variability in time and type of reactions to the same taste stimulus. We suppose that these different reactions may be classifiable into specific groups; however, such classification would require a larger sample size.

Our study considered a range of measures, and we found that right eyebrow elevation, left eyebrow elevation, and left mouth corner were the three most significant indicators of children’s taste perceptions, ranked in decreasing order of importance. Asymmetry in facial reactions has been observed elsewhere when considering emotional responses [48–50], and previous studies have found that the left side of the face expresses greater emotion than the right [49,50]. It should be noted that in our study the description of left and right is from the point of view of the observer of the videos, so our finding of the right eyebrow elevation as the most significant measure correlates with the left side of the child’s face showing a greater response. However, we also observed that many measures exhibited symmetry between their left and right variants.

Previous studies have also found the eyebrow lifting or lowering [45] and the mouth corners [45,46] to be present in response to bitter, sweet, and sour tastes. Wendin et al. [46] also identified frowning, wrinkled nose and eyes widening or squinting as present in sampling of bitter tastes. As our analysis considers variability of the facial measures, rather than absolute values, and given the black-box nature of the machine learning methods used, it is difficult to assign a single, specific facial reaction to any of the tastes. However, previous studies have reported that there is much overlap between responses, and it is indeed the combination of numerous factors that are associated with different tastes, rather than any individual change. For example, Wendin et al. [46] found that while sour elicited a greater response than bitter and sweet, frowning, wrinkling the nose, eye-widening and eye-diminishing were observed with both bitter and sour tastes. A study looking at the responses to different tastes in newborns found that the measures in the top and middle part of the face were similar across the tastes, while they differed in the lower component [15]. Future studies could therefore be improved by increasing the number and variety of facial measures included. For example, incorporating nose and forehead.

Eyebrow elevation was also slightly more variable in the tasting of strips that children rated with a low hedonic score compared to those that they rated more positively (Fig 7). Lower lip elevation showed a similar trend. Upper lip elevation has previously been found to be indicative of unpleasant taste [45] and in this current study, while showing less of a downward trend, did have a greater variability for the lowest scoring taste compared to the highest scoring. Weiland et al. [45] also found depression of the lip corners and lowering of the brow to be indicators of unpleasant tastes, which is supported here by the increase in variability of the corresponding measures as the hedonic score decreases. The results from the hedonic scale ratings support the methodology as the current gold standard for use with this age and ability group. However, further insight may be possible by analysing the facial reactions in relation to the hedonic ratings, for example, considering separately the reactions of those who liked and disliked the sour taste.

One limitation of this study is that the sample size obtained was smaller than what initially expected, although slightly larger than that of Kearns’ and Peng’s [39,40]. This can partly be explained by the fact that the study required proactive commitment from the caregiver’s side and the fact that several parents were not willing to provide video records of the face of their children, although clear explanation about privacy and data confidentiality were provided in the participant information sheet.

As discussed above, a second limitation of this study was that only selected facial expressions were considered. To generalise the findings, larger sample size and more diverse facial expression should be examined in future studies.

The results of this study provide new insights into the dynamics of facial expressions in response to taste stimuli. The study generated meaningful results, despite the relative lack of consistency and standardisation inherent in data gathered from a home setting, supporting the potential use of a decentralised approach. Further investigations could be conducted to explore other non-verbal cues to provide a more comprehensive understanding of the factors that influence taste perception in children. For instance, voice patterns may reveal vocal cues that indicate preferences or aversions. Besides that, body movement and hand gesture analysis can also offer valuable information on the emotional and cognitive responses to taste stimuli.

## Conclusion

Our study provides valuable insights into the complex nature of taste perception in children. Ensuring that orally administered medications are palatable is crucial for ensuring patients’ willingness to take them and comply with treatment. Despite limitations such as a smaller-than-expected sample size and the focus on selected facial expressions, our study provides valuable insights into taste perception dynamics in children. Capturing children’s objective responses to taste has important potential applications in supporting drug product development and measuring the acceptance of orally administered medications as part of clinical trials and longer-term adherence studies. While our methodology focused on verbal children, it lays the groundwork for future applications in populations unable to self-report their perceptions. The taste strips were easy to use and elicited expected results on the hedonic scale, reflecting children’s biological preferences for sweetness and aversion to bitterness. These findings may be used to develop interventions that enhance the understanding and acceptability of medications in children, ultimately to improve their overall health outcomes.

## References

[1] European Medicines Agency (EMA). Guideline on Pharmaceutical Development of Medicines for Paediatric Use (EMA/CHMP/QWP/805880/2012 Rev. 2). 2012 [cited 12 Dec 2020]. Available: https://www.ema.europa.eu/en/documents/scientific-guideline/guideline-pharmaceutical-development-medicines-paediatric-use_en.pdf

[2] European Medicines Agency (EMA) Committee for Medicinal Products for Human Use (CHMP). Reflection Paper: Formulations of Choice for the Paediatric Population. 2006 [cited 19 May 2023]. Available: https://www.ema.europa.eu/en/documents/scientific-guideline/reflection-paper-formulations-choice-paediatric-population_en.pdf

[3] Ternik R, Liu F, Bartlett JA, Khong YM, Thiam Tan DC, Dixit T, et al. Assessment of swallowability and palatability of oral dosage forms in children: Report from an M-CERSI pediatric formulation workshop. Int J Pharm. 2018;536: 570–581. doi: 10.1016/j.ijpharm.2017.08.088 28844897

[4] Kozarewicz P. Regulatory perspectives on acceptability testing of dosage forms in children. Int J Pharm. 2014;469: 245–248. doi: 10.1016/j.ijpharm.2014.03.057 24704104

[5] Davies EH, Tuleu C. Medicines for Children: A Matter of Taste. J Pediatr. 2008;153: 599–604.e2. doi: 10.1016/j.jpeds.2008.06.030 18940350

[6] Forestell CA, Mennella JA. The Ontogeny of Taste Perception and Preference Throughout Childhood. Handbook of Olfaction and Gustation. Hoboken, NJ, USA: John Wiley & Sons, Inc; 2015. pp. 795–828. doi: 10.1002/9781118971758.ch36

[7] Mennella JA, Spector AC, Reed DR, Coldwell SE. The bad taste of medicines: overview of basic research on bitter taste. Clin Ther. 2013;35: 1225–46. doi: 10.1016/j.clinthera.2013.06.007 23886820 PMC3772669

[8] Abdel-Rahman SM, Bai S, Porter-Gill PA, Goode GA, Kearns GL. A Pilot Comparison of High- Versus Low-Tech Palatability Assessment Tools in Young Children. Pediatric Drugs. 2021;23: 95–104. doi: 10.1007/s40272-020-00430-2 33236188

[9] Armstrong JE, Hutchinson I, Laing DG, Jinks AL. Facial Electromyography: Responses of Children to Odor and Taste Stimuli. Chem Senses. 2007;32: 611–621. doi: 10.1093/chemse/bjm029 17510090

[10] Shin IH, Park DC, Kwon C, Yeo SG. Changes in Taste Function Related to Obesity and Chronic Otitis Media With Effusion. Arch Otolaryngol Head Neck Surg. 2011;137: 242. doi: 10.1001/archoto.2011.23 21422307

[11] Sikka K, Ahmed AA, Diaz D, Goodwin MS, Craig KD, Bartlett MS, et al. Automated Assessment of Children’s Postoperative Pain Using Computer Vision. Pediatrics. 2015;136: e124–e131. doi: 10.1542/peds.2015-0029 26034245 PMC4485009

[12] Steiner JE. Facial Expressions Of The Neonate Infant Indicating The Hedonics Of Food-Related Chemical Stimuli. In: Weiffenbach JM, editor. Taste and development: The genesis of sweet preference. 1977. pp. 173–178. Available: https://books.google.co.uk/books?hl=it&lr=&id=P0JCgGAlwLkC&oi=fnd&pg=PA173&ots=KS2zTMxJ6D&sig=DD8HbsP0gcD4tkxYeHaj10-dl6A&redir_esc=y#v=onepage&q&f=false

[13] Steiner JE, Glaser D, Hawilo ME, Berridge KC. Comparative expression of hedonic impact: affective reactions to taste by human infants and other primates. Neurosci Biobehav Rev. 2001;25: 53–74. doi: 10.1016/s0149-7634(00)00051-8 11166078

[14] Forestell CA, Mennella JA. Early Determinants of Fruit and Vegetable Acceptance. Pediatrics. 2007;120: 1247–1254. doi: 10.1542/peds.2007-0858 18055673 PMC2268898

[15] Rosenstein D, Oster H. Differential Facial Responses to Four Basic Tastes in Newborns. Child Dev. 1988;59: 1555. doi: 10.2307/1130670 3208567

[16] Ekman P, Wallace V. F. Facial action coding system. Environmental Psychology & Nonverbal Behavior. 1978.

[17] Mennella JA, Forestell CA, Morgan LK, Beauchamp GK. Early milk feeding influences taste acceptance and liking during infancy. Am J Clin Nutr. 2009;90: 780S–788S. doi: 10.3945/ajcn.2009.27462O 19605570 PMC3136007

[18] Forestell CA, Mennella JA. More than just a pretty face. The relationship between infant’s temperament, food acceptance, and mothers’ perceptions of their enjoyment of food. Appetite. 2012;58: 1136–1142. doi: 10.1016/j.appet.2012.03.005 22407135 PMC3340480

[19] Guo X, Zhang Y, Lu S, Lu Z. Facial expression recognition: a review. Multimed Tools Appl. 2023;83: 23689–23735. doi: 10.1007/s11042-023-15982-x

[20] Leong SC, Tang YM, Lai CH, Lee CKM. Facial expression and body gesture emotion recognition: A systematic review on the use of visual data in affective computing. Comput Sci Rev. 2023;48: 100545. doi: 10.1016/j.cosrev.2023.100545

[21] De Sario GD, Haider CR, Maita KC, Torres-Guzman RA, Emam OS, Avila FR, et al. Using AI to Detect Pain through Facial Expressions: A Review. Bioengineering. 2023;10: 548. doi: 10.3390/bioengineering10050548 37237618 PMC10215219

[22] Nagireddi JN, Vyas AK, Sanapati MR, Soin A, Manchikanti L. The Analysis of Pain Research through the Lens of Artificial Intelligence and Machine Learning. Pain Physician. 2022;25: E211–E243. Available: http://www.ncbi.nlm.nih.gov/pubmed/35322975 35322975

[23] Webster PJ, Wang S, Li X. Review: Posed vs. Genuine Facial Emotion Recognition and Expression in Autism and Implications for Intervention. Front Psychol. 2021;12. doi: 10.3389/fpsyg.2021.653112 34305720 PMC8300960

[24] Landowska A, Karpus A, Zawadzka T, Robins B, Erol Barkana D, Kose H, et al. Automatic Emotion Recognition in Children with Autism: A Systematic Literature Review. Sensors. 2022;22: 1649. doi: 10.3390/s22041649 35214551 PMC8875834

[25] Staff AI, Luman M, van der Oord S, Bergwerff CE, van den Hoofdakker BJ, Oosterlaan J. Facial emotion recognition impairment predicts social and emotional problems in children with (subthreshold) ADHD. Eur Child Adolesc Psychiatry. 2022;31: 715–727. doi: 10.1007/s00787-020-01709-y 33415471 PMC9142461

[26] Turcian D, Stoicu-Tivadar V. Real-Time Detection of Emotions Based on Facial Expression for Mental Health. 2023. doi: 10.3233/SHTI230795 37869856

[27] Miyagi Y, Hata T, Bouno S, Koyanagi A, Miyake T. Recognition of facial expression of fetuses by artificial intelligence (AI). J Perinat Med. 2021;49: 596–603. doi: 10.1515/jpm-2020-0537 33548168

[28] De Risi M, Di Gennaro G, Picardi A, Casciato S, Grammaldo LG, D’Aniello A, et al. Facial emotion decoding in patients with Parkinson’s disease. International Journal of Neuroscience. 2018;128: 71–78. doi: 10.1080/00207454.2017.1366475 28796560

[29] Novotny M, Tykalova T, Ruzickova H, Ruzicka E, Dusek P, Rusz J. Automated video-based assessment of facial bradykinesia in de-novo Parkinson’s disease. NPJ Digit Med. 2022;5: 98. doi: 10.1038/s41746-022-00642-5 35851859 PMC9293947

[30] Alvarez VM, Dominguez-Soberanes J, Sanchez CN, Gutierrez S, Lopez B, Quiroz R, et al. Consumer Acceptances Through Facial Expressions of Encapsulated Flavors Based on a Nanotechnology Approach. 2018 Nanotechnology for Instrumentation and Measurement (NANOfIM). IEEE; 2018. pp. 1–5. doi: 10.1109/NANOFIM.2018.8688613

[31] Danner L, Sidorkina L, Joechl M, Duerrschmid K. Make a face! Implicit and explicit measurement of facial expressions elicited by orange juices using face reading technology. Food Qual Prefer. 2014;32: 167–172. doi: 10.1016/j.foodqual.2013.01.004

[32] Dibeklioglu H, Gevers T. Automatic Estimation of Taste Liking Through Facial Expression Dynamics. IEEE Trans Affect Comput. 2020;11: 151–163. doi: 10.1109/TAFFC.2018.2832044

[33] Samant SS, Chapko MJ, Seo H-S. Predicting consumer liking and preference based on emotional responses and sensory perception: A study with basic taste solutions. Food Research International. 2017;100: 325–334. doi: 10.1016/j.foodres.2017.07.021 28873694

[34] Yamamoto T, Mizuta H, Ueji K. Analysis of facial expressions in response to basic taste stimuli using artificial intelligence to predict perceived hedonic ratings. Li Z, editor. PLoS One. 2021;16: e0250928. doi: 10.1371/journal.pone.0250928 33945568 PMC8096070

[35] Zeinstra GG, Koelen MA, Colindres D, Kok FJ, de Graaf C. Facial expressions in school-aged children are a good indicator of ‘dislikes’, but not of ‘likes.’ Food Qual Prefer. 2009;20: 620–624. doi: 10.1016/j.foodqual.2009.07.002

[36] Zhi R, Cao L, Cao G. Asians’ Facial Responsiveness to Basic Tastes by Automated Facial Expression Analysis System. J Food Sci. 2017;82: 794–806. doi: 10.1111/1750-3841.13611 28140464

[37] Zhi R, Hu X, Wang C, Liu S. Development of a direct mapping model between hedonic rating and facial responses by dynamic facial expression representation. Food Research International. 2020;137: 109411. doi: 10.1016/j.foodres.2020.109411 33233098

[38] Rathod M, Dalvi C, Kaur K, Patil S, Gite S, Kamat P, et al. Kids’ Emotion Recognition Using Various Deep-Learning Models with Explainable AI. Sensors. 2022;22: 8066. doi: 10.3390/s22208066 36298415 PMC9607169

[39] Kearns GL, Bai S, Porter-gill PA, Goode GA, Farrar HC, Children A, et al. Use of facial recognition technology to assess drug palatability in pediatric patients: a pilot study. Journal of Applied Biopharmaceutics and Pharmacokinetics. 2019; 37–49. Available: https://www.scientificarray.org/wp-content/uploads/2020/02/JABPV7A5-Kearns.pdf

[40] Peng Y, Zhang H, Gao L, Wang X, Peng X. Palatability Assessment of Carbocysteine Oral Solution Strawberry Taste Versus Carbocysteine Oral Solution Mint Taste: A Blinded Randomized Study. Front Pharmacol. 2022;13. doi: 10.3389/fphar.2022.822086 35295331 PMC8919395

[41] Mueller C, Kallert S, Renner B, Stiassny K, Temmel AFP, Hummel T, et al. Quantitative assessment of gustatory function in a clinical context using impregnated “taste strips". Rhinology. 2003;44: 2–6. 12677732

[42] Lugaresi C, Tang J, Nash H, McClanahan C, Uboweja E, Hays M, et al. MediaPipe: A Framework for Building Perception Pipelines. 2019.

[43] Bazarevsky V, Kartynnik Y, Vakunov A, Raveendran K, Grundmann M. BlazeFace: Sub-millisecond Neural Face Detection on Mobile GPUs. 2019. Available: http://arxiv.org/abs/1907.05047

[44] Kartynnik Y, Ablavatski A, Grishchenko I, Grundmann M. Real-time Facial Surface Geometry from Monocular Video on Mobile GPUs. 2019. Available: http://arxiv.org/abs/1907.06724

[45] Weiland R, Ellgring H, Macht M. Gustofacial and Olfactofacial Responses in Human Adults. Chem Senses. 2010;35: 841–853. doi: 10.1093/chemse/bjq092 20876392

[46] Wendin K, Allesen-Holm BH, Bredie WLP. Do facial reactions add new dimensions to measuring sensory responses to basic tastes? Food Qual Prefer. 2011;22: 346–354. doi: 10.1016/j.foodqual.2011.01.002

[47] Yoshioka M, Ono Y, Komasa Y. Objective evaluation method of taste stimulation with pupil response. J Osaka Dent Univ. 2011; 111–119. 10.18905/jodu.45.1_111

[48] Delor B, D’Hondt F, Philippot P. The Influence of Facial Asymmetry on Genuineness Judgment. Front Psychol. 2021;12. doi: 10.3389/fpsyg.2021.727446 34899469 PMC8655228

[49] Lindell A. Lateralization of the expression of facial emotion in humans. 2018. pp. 249–270. doi: 10.1016/bs.pbr.2018.06.005 30097194

[50] Sackeim HA, Gur RC, Saucy MC. Emotions Are Expressed More Intensely on the Left Side of the Face. Science (1979). 1978;202: 434–436. doi: 10.1126/science.705335 705335

[51] Georgopoulos D, Keeley A, Tuleu C. Assessing the feasibility of using “taste-strips” for bitterness taste panels. Formulating Better Medicines for Children. Malmo; 2019. Available: https://www.ucl.ac.uk/pharmacy/sites/pharmacy/files/eupfi_taste-strips_poster.pdf

[52] Ranmal SR, Nhouchi Z, Keeley A, Adler L, Lavarde M, Pensé-Lhéritier A-M, et al. Taste assessment for paediatric drug Development: A comparison of bitterness taste aversion in children versus Naïve and expert young adult assessors. Int J Pharm. 2023;647: 123494. doi: 10.1016/j.ijpharm.2023.123494 37806503

[53] Mennella JA, Bobowski NK. The sweetness and bitterness of childhood: Insights from basic research on taste preferences. Physiol Behav. 2015;152: 502–507. doi: 10.1016/j.physbeh.2015.05.015 26002822 PMC4654709

[54] Liem DG. Heightened Sour Preferences During Childhood. Chem Senses. 2003;28: 173–180. doi: 10.1093/chemse/28.2.173 12588738 PMC2789429

[55] Liem DG, Mennella JA. Sweet and sour preferences during childhood: Role of early experiences. Dev Psychobiol. 2002;41: 388–395. doi: 10.1002/dev.10067 12430162 PMC2784884

